# Descriptive epidemiology of the first wave of COVID-19 in Petaling District, Malaysia: Focus on asymptomatic transmission

**DOI:** 10.5365/wpsar.2020.11.4.001

**Published:** 2021-04-21

**Authors:** Rama Krishna Supramanian, Lavanyah Sivaratnam, Arifah Abd Rahim, Noor Dalila Inche Zainal Abidin, Ong Richai, Zazarida Zakiman, Salina Md Taib, Lee Soo, Syed Hafeez Syed Ibrahim Jamalullai, Muhammad Nur Asraf Khirusalleh, Mohamed Paid Yusof

**Affiliations:** a Petaling District Health Office, Selangor, Malaysia.

## Abstract

**Background:**

COVID-19 was first detected in Malaysia on 25 January 2020. Multiple clusters were detected in Petaling District, with the first locally transmitted case reported on 8 February. Descriptive analyses of the epidemiology of the COVID-19 outbreak in Petaling are presented, from the first case to the end of the first wave.

**Methods:**

All laboratory-confirmed COVID-19 cases reported to the Petaling District Health Office between 1 February and 26 June 2020 were analysed. Socio-demographic characteristics, symptoms, date of onset, date of exposure, travel history and history of comorbidities were obtained by phone interviews using one of two investigation forms. The descriptive analysis was conducted according to time, place and person.

**Results:**

There were 437 COVID-19 cases, for an incidence rate of 24/100 000 population. Ten (2.3%) deaths and 427 recovered cases were recorded. Of the 437 cases, 35.5% remained asymptomatic and 64.5% were symptomatic. Common symptoms included fever (43.8%), cough (31.6%) and sore throat (16.2%); 67.3% had no comorbidities, 62.5% reported close contact with a confirmed case, and 76.7% were local infections. Transmission occurred in four main groups: religious gatherings (20.4%), corporations (15.1%), health facilities (10.3%) and a wholesale wet market (6.4%). In 31.9% of confirmed cases, an epidemiological link to an asymptomatic case was found.

**Conclusion:**

Transmission of the disease by asymptomatic cases should be emphasized to ensure continuous wearing of face masks, hand hygiene and social distancing. Further research should be conducted to better understand the transmission of SARS-CoV-2 from asymptomatic cases.

Pneumonia caused by severe acute respiratory syndrome coronavirus 2 (SARS-CoV-2) in Wuhan, China was first reported to the World Health Organization (WHO) on 31 December 2019. (*1*) As of 16 August 2020, the virus, which causes coronavirus disease 2019 (COVID-19), had spread globally and infected more than 21 million people, with more than 700 000 deaths. (*2*) The outbreak of COVID-19 was declared a public health emergency of international concern by the WHO on 30 January 2020, following international spread of the disease. Malaysia’s preparedness and response plan was instituted as early as February 2020. It included public health activities, intensified diagnostic capacity and early, appropriate treatment of confirmed COVID-19 cases. (*3*) The first cases of COVID-19 in Malaysia were detected on 25 January 2020 in three travellers from China, (*4*) and the first case in a Malaysian citizen was confirmed as the ninth case in early February 2020. (*4*) Localized clusters started to emerge in February, the largest cluster being linked to a religious gathering in Sri Petaling, which resulted in a major increase in the number of local cases and contributed to imported cases in neighbouring countries. (*5*) By 16 March, every state and federal territory in Malaysia had reported cases of COVID-19. Malaysia implemented a movement control order (MCO) on 18 March 2020 to contain the spread of the virus. (*4*) The government initiative included closing international borders, shutting down certain economic sectors and restricting social movement within and between states to protect the population. (*6*)

Many of the initial confirmed cases were connected to a wet market in Wuhan, and the SARS-CoV-2 pathogen was indicated to be zoonotic in origin. Reports have confirmed person-to-person transmission via respiratory droplets, as the virus was shown to spread in Wuhan by close contact with positive cases, without exposure to live animals. (*7*) The average incubation period for COVID-19 is 5 days but may be up to 14 days. The common reported symptoms include fever, cough, shortness of breath, fatigue and other flu-like symptoms. (*8*, *9*) Asymptomatic cases have also been documented. (*10*)

The first COVID-19 case in Petaling was documented on 3 February 2020 and was later confirmed to be the first case in a Malaysian citizen. (*4*) Following notification of a confirmed COVID-19 case, the District Health Office (DHO) implements control and prevention measures and conducts a thorough epidemiological investigation to identify the source of infection or index case. To break the chain of transmission, confirmed COVID-19 cases are isolated and treated in designated COVID-19 hospitals, while contacts are traced and identified for mandatory COVID-19 laboratory testing and a 14-day at-home quarantine. Early detection of cases among close contacts is crucial for early containment to prevent further seeding of community transmission. In early March 2020, the number of cases in Petaling increased due to a localized cluster of COVID-19 infections in one corporation, with more than 90 cases confirmed within 3 weeks. (*11*) The outbreak then increased exponentially, triggering a more rigorous control response from the Petaling DHO. As analysis of the COVID-19 cases in Petaling may provide critical information to help control the spread of similar infectious diseases at district and national levels, the objective of this paper is to describe the epidemiology of the COVID-19 epidemic in Petaling District between 1 February and 26 June 2020.

## Methods

### Study design

This descriptive study is based on an exploratory analysis of all cases of COVID-19 notified to the Petaling DHO from the beginning of the outbreak in early February 2020 to the end of June 2020.

### Study setting

This study was conducted in the Petaling District, Selangor, Malaysia, a highly urbanized area with a dense population of over 2 million people.

### Case definition

The definition of a confirmed case of COVID-19 is a person with a reverse transcriptase polymerase chain reaction (RT–PCR) positive result, regardless of their symptoms. Only cases that met this case definition were included. People under investigation for COVID-19 are defined as having fever OR acute respiratory infection (sudden onset of shortness of breath, cough and/or sore throat) AND travel to or residence in an affected country (China, Islamic Republic of Iran, Italy, Japan, Republic of Korea) within 14 days before illness onset OR close contact within 14 days before illness onset with a confirmed case of COVID-19.

### Epidemiological investigation

Each notified case was verified by the Petaling DHO before an epidemiological investigation was undertaken to determine the source of infection, including contact tracing, active case detection and prevention and control measures, including quarantine. The primary objective of investigation was to identify the source of infection and close contacts of confirmed cases. Information on socio-demographic characteristics, symptoms, date of illness onset, date of exposure, travel history and comorbidities was obtained by phone interview with cases and contacts using one of two investigation forms. The data were then shared with the Selangor State Health Department. Date of exposure was defined as the last date of contact with a known case of COVID-19 or last date of travel, if any, while date of onset was defined as the date the person self-reportedly developed any symptoms related to COVID-19. Details of close contacts were retrieved during case investigations and sent to the contact tracing team for further action. All cases and contacts were monitored daily for the next 14 days. All relevant data were captured within the COVID-19 surveillance system of Malaysia’s Ministry of Health (health ministry).

### Data management

Most notifications of confirmed cases were received from the Surveillance Unit of the Selangor State Health Department; some were received by phone, fax or e-mail from hospitals and accredited laboratories. The health ministry has a surveillance system for notification and monitoring of infectious diseases known as the Communicable Diseases Control Information System or eNotifikasi, (*12*) and COVID-19 was added as a notifiable disease to this system at the end of March 2020 to ensure mandatory reporting of suspected and confirmed cases of COVID-19 to the nearest DHO. Reporting is compulsory under the Malaysia Prevention and Control of Infectious Disease Act 1988. (*13*) As all case records contain national identification numbers, all cases are recorded in the system without duplication. The inclusion criteria for this study were confirmed COVID-19 cases according to the case definition notified to the Petaling DHO between 1 February 2020 and 26 June 2020.

### Data analysis

The socio-demographic and clinical characteristics of all confirmed cases of COVID-19 were summarized with descriptive statistics. An epidemic curve of all cases was constructed by plotting the number of cases (*y*-axis) against the self-reported date of symptom onset (*x*-axis). For asymptomatic cases, the date of onset was considered to be the last date of known exposure.

### Ethical approval

The study protocol was approved by the Medical Research and Ethics Committee, health ministry Malaysia (NMRR-20–1540–55803 [IIR]).

## Results

Between 1 February 2020 and 26 June 2020, there were 437 confirmed cases of COVID-19 in Petaling District. The total population of Petaling District in the 2010 census (*14*) was 1 812 633. Therefore, the incidence rate of COVID-19 infection was 24/100 000 population. The baseline characteristics of the confirmed cases are presented in **Table 1**.

**Table 1:** Baseline characteristics of COVID-19 cases in Petaling District

-	N	%
**Total number of cases**	**437**	
**Attack rate**	**-**	**0.024**
**Age (years, mean, SD)**	**41, 17.7**	
Age group		
0–10	15	3.4
11–20	28	6.4
21–30	112	25.6
31–40	77	17.6
41–50	59	13.5
51–60	77	17.6
> 60	69	15.8
Gender		
Male	235	53.8
Female	202	46.2
Nationality		
Malaysian	402	92.0
Non-Malaysian	35	8.0
Symptom status		
Symptomatic	282	64.5
Asymptomatic	155	35.5
Symptoms (*n* = 282)		
Fever	187	43.8
Cough	137	31.6
Sore throat	71	16.2
Headache	22	5.0
Loss of taste and smell	21	4.8
Myalgia	18	4.1
Gastrointestinal disturbances	12	2.7
Comorbidities or risk factors		
None	294	67.3
Hypertension	70	16.0
Diabetes mellitus	46	10.6
Dyslipidaemia	22	5.0
Heart disease	16	3.7
Bronchial asthma	10	2.3
History of close contact with a confirmed COVID-19 case		
Yes	273	62.5
No	164	37.5
Total number of close contacts		
Symptomatic index cases	4568	64.5
Asymptomatic index cases	2513	35.5
Confirmed COVID-19 cases among close contacts		
Symptomatic index cases	109	68.1
Asymptomatic index cases	51	31.9
Type of infection		
Local	335	76.7
Imported	102	23.3
Clusters		
Religious gathering	89	20.4
Corporation	66	15.1
Health facilities	45	10.3
Wholesale wet market	28	6.4
Others	209	47.8
Local council area subdivision		
Petaling Jaya	178	40.7
Subang Jaya	100	22.9
Shah Alam	157	36.0
Others	2	0.5
Status		
Alive	427	97.7
Dead	10	2.3

All 437 cases were admitted to the hospital for isolation and treatment. Ten cases (2.3%) died due to complications, and the other 427 cases were eventually discharged. Of all cases, 76.7% were local and 23.3% were imported. The mean age was 41 years, and 25.6% were in the 21–30 years age group. The gender distribution was relatively even, with 53.8% male and 46.2% female cases. Malaysian citizens accounted for 92%, and 64.5% of cases were symptomatic. The most commonly observed symptoms were fever (43.8%), cough (31.6%) and sore throat (16.2%). The total number of close contacts of confirmed COVID-19 cases was 7081. Among 160 close contacts who were later confirmed positive, 51 (31.9%) were close contacts of asymptomatic primary cases, and 109 (68.1%) were close contacts of symptomatic primary cases.

A total of 294 cases (67.3%) had no comorbidities, while 70 (16%) had hypertension and 46 (10.6%) had diabetes mellitus. Of all cases, 62.5% had reported close contact with a confirmed COVID-19 case, and 76.7% were classified as locally transmitted infections. In Petaling, four main clusters of cases were identified: at a religious gathering (20.4%), in a corporation (15.1%), in health facilities (10.3%) and at a wholesale wet market (6.4%). Other clusters included sporadic local and imported cases.

**Figure 1:**
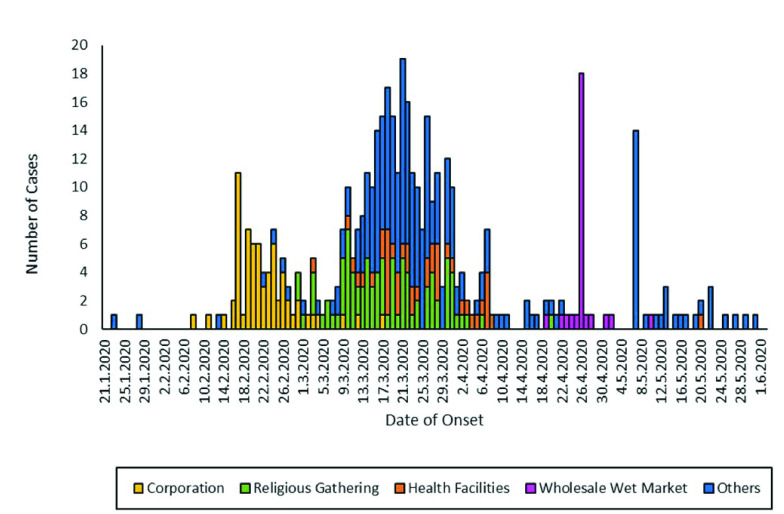
COVID-19 epidemic curve, with all clusters in Petaling District between January and June 2020

**Fig. 1** shows the dates of symptom onset for cases of COVID-19 in Petaling District between January and June 2020. The first cluster of COVID-19 was detected in a corporation in early February, which peaked in mid-February. A total of 66 cases were reported from this cluster. The highest peak of cases occurred in mid-March; the infection rate then tapered off and ended in mid-April. Most cases during the peak were linked to a mass religious gathering (89 cases). The third peak, seen at the end of April, involved vendors at a wholesale wet market, with a total of 28 cases reported. The epidemic curve in **Fig. 1** shows a pattern indicating person-to-person transmission.

[**insert** Figure 1]

## Discussion

We report the epidemiological characterization of the initial COVID-19 outbreak in the most densely populated district of the State of Selangor, Malaysia. Most of the reported cases were aged 21–30 years (25.6%), and the distribution of cases by gender was similar. The age distribution of the cases in this study is consistent with that in the initial outbreak reported in China, i.e. mainly young adults. (*15*) About 65% of the cases were symptomatic, the three most commonly reported symptoms being fever, cough and sore throat. The pathogenesis of SARS-CoV-2 includes both upper and lower respiratory tract infections, (*16*) and the earliest outbreak in the epicentre, Wuhan, also included symptoms of respiratory tract infection in most reported cases. (*17*) Respiratory viruses are highly contagious when patients are symptomatic. In the outbreak reported here, more than half the cases were locally transmitted and had reported close contact with a confirmed COVID-19 case. COVID-19 is transmitted primarily in respiratory droplets (*7*) and by physical contact. (*18*) Evidence of human-to-human transmission among close contacts has been found since the beginning of the pandemic, in mid-December 2019. (*19*) Furthermore, the clusters of COVID-19 cases in Petaling District involved gatherings, further spreading the virus in the community. (*20*) In this outbreak, about 32% of cases had an epidemiological link to an asymptomatic case. As similar viral loads have been reported in symptomatic and asymptomatic cases, (*17*) community transmission of SARS-CoV-2 by asymptomatic cases is likely. The salivary glands could act as a potential reservoir for COVID-19; thus, infectious salivary droplets could be transmitted to a susceptible host from the mouth when an asymptomatic carrier is speaking, sneezing or even breathing, or from the eyes, and directly inhaled into the lungs. (*21*) Similar evidence of transmission from asymptomatic carriers to close contacts has been reported. (*22*, *23*) In view of the novelty of SARS-CoV-2, accumulation of evidence on transmission from asymptomatic people has contributed to understanding the dynamics and public health implications of the disease. In our study, almost one third of close contacts who became infected were contacts of asymptomatic cases. As asymptomatic individuals appear to be a common source of infection, strict monitoring of close contacts of asymptomatic cases is essential to contain potential outbreaks.

The fundamental characteristics of first-wave cases and the associated epidemic curves in Petaling District indicate that 282 (64.5%) cases were symptomatic, with appropriate dates of onset of illness. The epidemic curve of all clusters in Petaling District between January and June 2020 (**Fig. 1**) indicates that the outbreak had a propagated source pattern of spread. This trend is consistent with person-to-person spread in outbreaks of this newly introduced zoonotic viral pathogen that subsequently became capable of human-to-human transmission due to high mutation and recombination rates. (*24*) As shown in the epidemic curve, the outbreak in Petaling District had multiple surges of cases, resulting from several main clusters, including a corporation, a religious gathering, health facilities, a wholesale wet market and sporadic cases. The index case in the corporation cluster was believed to have been infected while travelling in Indonesia before the onset of symptoms. Subsequently, while symptomatic, the index case attended a meeting at the office, and transmission occurred to other workers. The religious gathering was attended by more than 19 000 people from various countries. It not only became a catalyst for subsequent spread of COVID-19 in Petaling District but also resulted in massive transmission throughout Malaysia and abroad. (*25*) The gathering involved sharing of communal spaces, such as prayer halls, collective eating from shared plates and sharing of sleeping areas, which increased the opportunities for transmission among participants. Transmission of COVID-19 in these two main reported clusters in Petaling District went beyond household contacts, and contact tracing revealed up to five generations of contacts. The epidemic curve shows that cluster transmission accounted for more than half of the confirmed COVID-19 cases in this outbreak; a similar phenomenon has been seen in other cities. (*18*)

Early implementation of the MCO in response to the COVID-19 pandemic played a vital role in controlling the outbreak and preventing disease transmission within the community. Closure of all universities, schools, places of worship and non-essential sectors during the MCO helped to break the chain of transmission in the community by prohibiting mass movement and gatherings nationwide. This federal response was successful in lowering the epidemic curve in Petaling District. The enhanced or targeted MCO, a cordon sanitaire implemented on 10 May 2020 by the federal government, slowed the COVID-19 outbreak in Petaling District during the wholesale wet market cluster.

Overall, this study provides valuable information on the first wave of the COVID-19 outbreak in Petaling District and the general epidemiological measures taken to curb the outbreak. Additionally, this study included a large number of cases, as Petaling is part of the state of Selangor, which had the second-largest number of confirmed COVID-19 cases in Malaysia during this period of the pandemic. Nevertheless, the study had some limitations, such as lack of data on the severity and clinical outcomes of cases. Furthermore, the data were retrospective and self-reported by patients and may be inaccurate due to recall bias.

## Conclusion

This study provides key findings in the Petaling COVID-19 outbreak that are consistent with those reported in other studies. Most cases had a history of close contact with confirmed COVID-19 cases, confirming human-to-human transmission. The study also confirms that asymptomatic cases can transmit the disease to others. This should be emphasized to the community to ensure continuous wearing of face masks, hand hygiene and social distancing in public. Public health efforts should focus on surveillance for local transmission of cases and swift control measures to avert widespread community transmission. Active case detection and quarantine of close contacts of confirmed cases is a key prevention and control strategy to prevent spread of the disease, while strict monitoring of close contacts of asymptomatic infected cases is just as important as for symptomatic cases. Further research should be conducted to better understand the transmission of SARS-CoV-2 from asymptomatic cases.
